# Validating the Rett Syndrome Gross Motor Scale

**DOI:** 10.1371/journal.pone.0147555

**Published:** 2016-01-22

**Authors:** Jenny Downs, Michelle Stahlhut, Kingsley Wong, Birgit Syhler, Anne-Marie Bisgaard, Peter Jacoby, Helen Leonard

**Affiliations:** 1 Telethon Kids Institute, The University of Western Australia, Perth, Australia; 2 School of Physiotherapy and Exercise Science, Faculty of Health Sciences, Curtin University, Perth, Australia; 3 Center for Rett Syndrome, Department of Clinical Genetics, Rigshospitalet, Copenhagen, Denmark; Institute of Genetics and Biophysics, ITALY

## Abstract

Rett syndrome is a pervasive neurodevelopmental disorder associated with a pathogenic mutation on the *MECP2* gene. Impaired movement is a fundamental component and the Rett Syndrome Gross Motor Scale was developed to measure gross motor abilities in this population. The current study investigated the validity and reliability of the Rett Syndrome Gross Motor Scale. Video data showing gross motor abilities supplemented with parent report data was collected for 255 girls and women registered with the Australian Rett Syndrome Database, and the factor structure and relationships between motor scores, age and genotype were investigated. Clinical assessment scores for 38 girls and women with Rett syndrome who attended the Danish Center for Rett Syndrome were used to assess consistency of measurement. Principal components analysis enabled the calculation of three factor scores: Sitting, Standing and Walking, and Challenge. Motor scores were poorer with increasing age and those with the p.Arg133Cys, p.Arg294* or p.Arg306Cys mutation achieved higher scores than those with a large deletion. The repeatability of clinical assessment was excellent (intraclass correlation coefficient for total score 0.99, 95% CI 0.93–0.98). The standard error of measurement for the total score was 2 points and we would be 95% confident that a change 4 points in the 45-point scale would be greater than within-subject measurement error. The Rett Syndrome Gross Motor Scale could be an appropriate measure of gross motor skills in clinical practice and clinical trials.

## Introduction

Rett syndrome is a neurodevelopmental disorder usually caused by a mutation on the X-linked methyl-CpG-binding protein 2 (*MECP2*) gene.[1] The condition affects females approximately 1 per 9,000 live female births,[2] and is characterized by a loss of functional hand use and language skills in early childhood with the development of hand stereotypies and impaired mobility.[3] These developmental issues are complicated by frequent occurrence of comorbid conditions. Recent large cross-sectional studies have found marked variability in phenotype in part explained by the type of genetic mutation.[4, 5] With regard to motor abilities, girls and women with mutation p.R270X or p.R168X present with a more severe phenotype, whereas those with p.R133C, p.R294X, and C-terminal deletions are more likely to walk.[4, 5]

Early clinical descriptions suggested that neurological impairments such as hypotonia and weakness in the early years and dystonia and bradykinesia in the later years[6] impacted motor function. It was also reported that the gait may be rigid with a lack of co-ordinated movements of the upper extremities and an unsteady wide base.[6] We previously found that most girls with Rett syndrome learn to sit and approximately half learn to walk during their early development.[7] Some individuals are able to maintain the ability to walk through adulthood,[8] but others develop bradykinesia and increased muscle tone, and with increasing difficulty maintaining upright postures, the ability to walk is lost.[6]

We previously adapted the Gross Motor Function Measure[9, 10] and included several additional items to form a smaller scale suitable for administration to those with Rett syndrome.[11] Families participating in the Australian Rett Syndrome Database (ARSD) were asked to video their daughter performing a set of functional activities and also complete a parallel parent-report checklist. Observing the videos, we classified each motor skill according to the amount of assistance required and demonstrated substantial to excellent inter-rater reliability for coding each of the items.[11] Using principal components analysis, 15 items were reduced to two subscales, one describing general motor skills and the other more complex gross motor skills.[12] This initial validation was promising: the factors were conceptually consistent with different aspects of motor function and we found a general decline in motor skill capacity with increasing age.[12] However, the ARSD is now custodian to a substantially larger dataset and additional examination of the measurement properties the Rett Syndrome Gross Motor Scale (RSGMS) is justified. Clear understanding of the measurement properties of scales appropriate to Rett syndrome is critical in this era of clinical trials for neurodevelopmental disorders.

Therefore, the objectives of the current study were to replicate a principal components analysis of the RSGMS using our current larger dataset and to assess relationships of motor scores with genotype and age. We also sought to investigate the consistency of observed and parent-reported ratings and to describe the test-retest and within-subject reliability of the RSGMS using clinical data collected at the Danish Rett Syndrome Center.

## Materials and Methods

### Australian Data

The Australian Rett Syndrome Database (ARSD) is a population-based register established in 1993 of confirmed individuals with Rett syndrome born 1976 and subsequently.[2, 13] Families/carers of females with Rett syndrome are invited to complete an initial questionnaire at the time of registration and follow-up surveys have been administered in 2000, 2002, 2004, 2006, 2009 and 2011. In 2004, 2007 and 2012 families were sent a filming protocol and a parent-report checklist, and asked to film their daughters’ everyday tasks in their familiar environment including listed activities to demonstrate gross motor function.[11] In 2004, families were also sent a blank video for data collection but with evolving technologies, DVDs and online data transfer methods were used in 2007 and 2012.

A total of 99 videos were collected in 2004, 178 in 2007 and 171 in 2012. As per previous methods,[8, 12] a trained researcher coded the gross motor items according to the observed level of assistance. Categories of assistance included no assistance, mild assistance, moderate assistance or maximal assistance/unable, using a 0 to 3 scale with 3 representing better function (See S1 Appendix). The most recently collected video data from each unique individual was used to replicate the principal components analysis. Gross motor items observed on the 2012 video and where available, classified by parents on the parallel parent-report checklist were used to assess the consistency of observed and parent-reported scores.

Data from the ARSD was used to describe the type of *MECP2* mutation and age. Individuals were categorized by *MECP2* mutation, including the C-terminal deletions, large deletion, early truncating, and p.Arg106Trp, p.Arg133Cys, p.Thr158Met, p.Arg168*, p.Arg255*, p.Arg270*, p.294* and p.Arg306Cys mutations. A final group included all other pathogenic mutations. Age was grouped into 4 categories representing the preschool and early school years (<8 years), primary school (8 < 13 years), adolescent (13 < 19 years), and adult (≥ 19) years.

### Danish Data

The National Center for Rett syndrome in Denmark was established in 2007 and offers counselling and annual follow-up by a multidisciplinary team. Currently, there are 109 known confirmed individuals (age two to 61 years) with Rett syndrome in Denmark of whom 96 (88%) have a *MECP2* mutation. Convenience sampling was used in this study comprising girls and women with Rett syndrome and a *MECP2* mutation who lived in the Capital Region or Region Zealand in Denmark. Thirty-nine participants were invited and 38 were assessed twice with the RSGMS approximately one week apart (one child was unavailable for the second assessment). Nine physiotherapists performed the assessments with each participant who was assessed by the same physiotherapist on both occasions. All physiotherapists had a background in pediatric physiotherapy with two to 25 years of work experience and were trained in the administration of the RSGMS. Assessments took place in the pre-school, school, day activity centre or in the home of the participants according to the preferences of the parents/caregivers. Thus, the assessments reflected the usual performance of the participants in their local environment.

Ethical approvals of this study were provided by the Human Research Ethics Committee at Princess Margaret Hospital for Children, Western Australia (1909EP) and The Regional Scientific Ethical Committee in the Capital Region of Denmark (H-6-2014-074). Written informed consent was obtained from parents or legal guardians on behalf of their child to participate in this study.

### Analyses

#### Australian data

Principal components analysis using varimax rotation was performed to reduce the set of 15 items into a smaller number of independent variables. Listwise deletion in principal components analysis has the disadvantage of markedly reducing the sample size because records with small numbers of missing items are ineligible. Pairwise deletion was chosen because it enables a maximal sample size.[14] Therefore, all video records where more than one skill was observed were included. A cutoff Eigenvalue of 0.9 was chosen after inspection of the scree plot to define the factors. The internal consistency of the factors was analysed using Cronbach’s α. Multiple quantile regression or logistic (for binary outcomes) regression models were used as appropriate to examine the association between motor scores and age and mutation groups. Using the 2012 dataset, chance-corrected agreements for each coding category as reported by the experienced assessor and parent-report were compared using Cohen’s Kappa statistic. As previously classified, Kappa coefficients above 0.8 were interpreted as excellent, 0.6–0.8 as substantial, 0.4–0.6 as moderate and below 0.4 as poor.[15] The observed score for each item was replaced by the parent-reported item score and the mean (SD) difference in total and subscale scores was calculated for observations with a full set of observed data.

#### Danish data

Intraclass correlation coefficients (ICC) were calculated. The standard error of measurement, defined as the square root of the mean square within subjects error term using repeated measures analysis of variance, was determined and then used to calculate the minimal detectable difference (SEM x 1.96 x √2).[16]

Principal components analysis was conducted using SPSS (IBM Corp. Released 2013. IBM SPSS Statistics for Windows, Version 22.0. Armonk, NY: IBM Corp.) and all other analyses were undertaken using Stata (Version 13, StataCorp, College Station, TX, USA).

## Results

### Validation

Video data were available for 255 individuals including 170 (66.7%) collected in 2012, 65 (25.5%) in 2007 and 20 (7.8%) in 2004. Table 1 shows the distribution of the sample by mutation type and age group.

**Table 1:** Frequency distribution of mutation type and age group categories for individuals included in the principal components and regression analyses.

		Sample included in the principal components analysis (N = 255)	Sample included in regression analyses (N = 215)
Common mutation, N (%)	C-terminal deletion	22 (8.6)	22 (10.2)
	Early truncating	15 (5.9)	15 (7.0)
	Large deletion	15 (5.9)	15 (7.0)
	p.Arg106Trp	12 (4.7)	12 (5.6)
	p.Arg133Cys	19 (7.5)	19 (8.8)
	p.Arg168*	23 (9.0)	23 (10.7)
	p.Arg255*	16 (6.3)	16 (7.4)
	p.Arg270*	19 (7.5)	19 (8.8)
	p.Arg294*	19 (7.5)	18 (8.8)
	p.Arg306Cys	11 (4.3)	11 (5.1)
	p.Thr158Met	19 (7.5)	19 (8.8)
	Other	25 (9.8)	25 (11.6)
	Negative	35 (13.7)	-
	Not tested	5 (2.0)	-
Age group, N (%)	< 8 years	47 (18.4)	45 (20.9)
	8 < 13 years	46 (18.0)	44 (20.5)
	13 < 19 years	59 (23.1)	48 (22.3)
	≥ 19 years	103 (40.4)	78 (36.4)

Principal components analysis of the 15 items resulted in the extraction of three factors that accounted for 82% of total data variance (Table 2). Nine items describing skills of sit to stand, standing, walking, side stepping, turning, walking on a slope and stepping over an obstacle loaded strongly on to factor one which was named ‘Standing and Walking’ because the items related to weight bearing activities (Eigenvalue 9.8 and accounting for 43.4% of the variance). Three items describing skills of moving from the floor to standing, picking up an object from the floor from standing and running loaded onto factor two and this was named ‘Challenge’ because of the complexity of the skills (Eigenvalue 1.5 and accounting for 20.9% of the variance). Three items describing sitting on the floor, on a chair and on a stool loaded onto factor three which was named ‘Sitting’ (Eigenvalue 0.92 and accounting for 17.5% of the variance). The Cronbach’s alpha coefficient for the total scale was 0.96, for ‘Standing and Walking’ 0.97, for ‘Challenge’ 0.85 and for ‘Sitting’ 0.83.

**Table 2:** Factor loadings for individual scale items onto each of the three factors.

Item	N	‘Sitting’ (Factor 3)	‘Standing and Walking’ (Factor 1)	‘Challenge’ (Factor 2)
Sitting on the floor	233	**0.694**	0.245	0.350
Sitting on a chair	250	**0.747**	0.493	0.077
Sitting on a stool	238	**0.844**	0.336	0.109
Sit to stand	240	0.305	**0.727**	0.157
Standing 3 s	252	0.361	**0.878**	0.183
Standing 10 s	249	0.356	**0.857**	0.452
Standing 20 s	241	0.326	**0.830**	0.197
Walks 10 steps	249	0.303	**0.891**	0.218
Side steps	234	0.272	**0.747**	0.405
Turns	242	0.279	**0.870**	0.293
Walking on a slope	220	0.213	**0.819**	0.353
Steps over an obstacle	226	0.185	**0.623**	0.515
Stands up from floor	228	0.285	0.359	**0.734**
Bends to touch the floor	233	0.123	0.194	**0.867**
Runs	243	0.069	0.195	**0.847**

The median (interquartile range [IQR]) was 10/45 (IQR 1, 28) for the total score and 3/27 (IQR 0, 20) for the Standing and Walking subscale. Not surprisingly, the median (IQR) score for the Sitting subscale of 6/9 (IQR 0, 20) was higher than that for the Challenge subscale of 0/9 (IQR 0, 1). As the majority of individuals were unable to attain better than minimal level of skills included in the Challenge subscale, the score was dichotomised into a binary outcome (0, ≥1). Multivariate relationships between motor scores, age group and mutation are shown in Table 3. In general, scores decreased with increasing age although not significantly for the total and Standing and Walking scores. Compared to children younger than eight years, teenagers received three points less for the Sitting subscale (95% confidence interval [CI] -6, 0; p = 0.038) as did adults (95%CI -6, 0; p = 0.021). Also compared to children younger than eight years, adults were 75% more likely to be unable to score on any of the Challenge subscale items (odds ratio [OR] 0.25; 95%CI 0.10, 0.65; p = 0.004) (Table 3).

**Table 3:** Relationships between total and subscale scores, age group and genotype.

Factor	Total score (N = 166)			Sitting score (N = 223)			Standing and Walking score (N = 176)			Challenge score (N = 191)		
	n	Coefficient^a^ (95%CI)	P value	n	Coefficient^a^ (95%CI)	P value	N	Coefficient^a^ (95%CI)	P value	n	OR^b^ (95%CI)	P value
Age group												
< 8 years	35	baseline	-	40	baseline	-	36	baseline	-	39	baseline	-
8 < 13 years	31	-2 (-14,10)	0.747	38	-1 (-4,2)	0.507	35	-2 (-11,7)	0.646	40	0.64 (0.23,1.74)	0.381
13 < 19 years	39	-4 (-15,7)	0.486	44	-3 (-6,0)	**0.038**	43	-2 (-10,6)	0.625	42	0.55 (0.20,1.51)	0.248
≥ 19 years	61	-8 (-18,2)	0.120	68	-3 (-6,0)	**0.021**	62	-3 (-10,4)	0.419	70	0.25 (0.10,0.65)	**0.004**
Mutation												
Large deletion	13	baseline	-	14	baseline	-	15	baseline	-	14	baseline	-
C-terminal deletion	18	10 (-7,27)	0.247	21	4 (0,8)	0.068	18	9 (-3,21)	0.139	18	1.85 (0.36,9.60)	0.462
Early truncating	13	1 (-17,19)	0.915	14	1 (-4,6)	0.678	13	0 (-13,13)	1.000	14	0.56 (0.08,4.18)	0.573
p.Arg106Trp	10	11 (-9,31)	0.278	10	2 (-3,7)	0.453	11	0 (-14,14)	1.000	12	1.70 (0.27,10.73)	0.575
p.Arg133Cys	13	21 (3,39)	**0.025**	13	4 (-1,9)	0.102	14	19 (6,32)	**0.003**	16	3.34 (0.64,17.33)	0.151
p.Arg168*	17	11 (-6,28)	0.212	20	4 (0,8)	0.073	18	7 (-5,19)	0.251	21	0.75 (0.13,4.20)	0.743
p.Arg255*	11	-1 (-20,18)	0.918	15	-1 (-6,4)	0.673	11	0 (-14,14)	1.000	15	0.99 (0.16,6.24)	0.992
p.Arg270*	16	0 (-18,18)	1.000	18	-1 (-5,3)	0.661	17	2 (-10,14)	0.748	17	1.09 (0.19,6.34)	0.924
p.Arg294*	11	24 (5,43)	**0.015**	17	4 (-1,9)	0.084	12	15 (2,28)	**0.027**	15	4.38 (0.82,23.37)	0.084
p.Arg306Cys	10	23 (3,43)	**0.022**	11	5 (0,10)	0.051	10	21 (7,35)	**0.003**	11	2.13 (0.35,12.93)	0.412
p.Thr158Met	14	5 (-13,23)	0.590	15	1 (-4,6)	0.677	15	3 (-10,16)	0.640	15	0.78 (0.12,5.03)	0.797
Other	20	13 (-4,30)	0.135	22	4 (0,8)	0.072	22	6 (-6,18)	0.313	23	2.31 (0.46,11.50)	0.308

^a^ Coefficient values represent change in the outcome in relation to the predictor relative to the median value of the baseline group.

^b^ Odds ratio refers to the odds of the outcome given exposure to the predictor variable.

Those with mutations such as a large deletion, p.Arg255* or p.Arg270* generally had lower scores than those with mutations such as p.Arg133Cys, p.Arg294* or p.Arg306Cys. Compared to those with the large deletion, those with the p.Arg133Cys (median 21; 95%CI 3, 39; p = 0.025), p.Arg294* (median 24; 95%CI 5, 43; p = 0.015) or p.Arg306Cys (median 23; 95%CI 3, 43; p = 0.022) mutation achieved higher total scores. Similarly and compared to those with the large deletion, those with the p.Arg133Cys (median 19; 95%CI 6, 32; p = 0.003), p.Arg294* (median 15; 95%CI 2, 28; p = 0.027) or p.Arg306Cys (median 21; 95%CI 7, 35; p = 0.003) mutation achieved higher Standing and Walking scores. Sitting scores were relatively more consistent across the mutation groups but compared to the large deletion, those with the p.Arg306Cys achieved five points more (95%CI 0, 10; p = 0.051). Challenge scores were low across the mutation groups but those with the p.Arg294* had more than four times the odds of achieving a score greater than 0 than those with a large deletion (95%CI 0.82, 23.37; p = 0.084). Those with the p.Arg294* consistently achieved high motor scores across the different scores (Table 3).

Comparing observed and parent-reported levels of assistance for each item, Kappa values indicated moderate to good agreement with values ranging from 0.47 for sit to stand to 0.75 for running (Table 4). Substitution of one observed score with a parent-reported score had minimal effects on the total and each of the subscale scores. The differences in scores were less than half of one point for each scale, ranging from -0.16 to 0.41 for the total score, -0.15 to 0.26 for the Standing and Walking subscale, -0.19 to 0.01 for the Challenge subscale and 0.17 to 0.42 for the Sitting subscale (Table 4).

**Table 4:** Kappa values for observed and parent-reported item scores and differences between observed scores and the modified observed scores when one item replaced with the parent reported score.

Item (n)	Kappa values^a^ (95% CI)	Mean (SD) difference in scores when 1 observed score replaced with the equivalent parent-report score			
		Total score (/45)	Sitting factor (/9)	Standing and Walking factor (/27)	Challenge factor (/9)
Sitting on the floor (112)	0.58 (0.44, 0.72)	0.04 (0.95)	0.17 (0.99)	-	-
Sitting on a chair (123)	0.51(0.40, 0.62)	0.33 (0.88)	0.26 (0.77)	-	-
Sitting on a stool (114)	0.53(0.41, 0.65)	0.41 (1.00)	0.42 (0.99)	-	-
Sit to stand (119)	0.47(0.37, 0.56)	-0.01 (0.62)	-	-0.03 (0.67)	-
Standing 3 s (123)	0.68 (0.55, 0.80)	0.11 (0.81)	-	0.08 (0.84)	-
Standing 10 s (124)	0.61(0.49, 0.73)	0.13 (0.84)	-	0.09 (0.86)	-
Standing 20 s (115)	0.72 (0.59, 0.85)	0.06 (0.71)	-	0.03 (0.76)	-
Walks 10 steps (99)	0.59 (0.47, 0.72)	0.31 (0.70)	-	0.25 (0.67)	-
Side steps (87)	0.64 (0.50, 0.79)	0.19(0.79)	-	0.26 (0.83)	-
Turns (97)	0.60 (0.47, 0.73)	0.17 (0.83)	-	0.15 (0.77)	-
Walking on a slope (80)	0.59 (0.46, 0.72)	0.21 (0.73)	-	0.19 (0.77)	-
Steps over an obstacle (83)	0.58 (0.45, 0.71)	-0.09 (0.78)	-	-0.15 (0.84)	-
Stands up from floor (112)	0.52 (0.40, 0.64)	0.01 (0.50)	-	-	0.01 (0.62)
Bends to touch the floor (112)	0.66 (0.51, 0.81)	-0.14 (0.61)	-	-	-0.19 (0.69)
Runs (88)	0.75 (0.58, 0.92)	-0.16 (0.56)	-	-	-0.11 (0.48)

^a^ Kappa coefficients above 0.8 were interpreted as excellent, 0.6–0.8 as substantial, 0.4–0.6 as moderate and below 0.4 as poor.

### Reliability

Thirty-eight girls and women participated in the test-retest analyses at a median (IQR) age of 16.9 (6.8, 34.7) years (S1 Table). All mutation categories were represented (C-terminal deletion [n = 4], early truncating [n = 3], large deletion [n = 4], p.Arg106Trp [n = 1], p.Arg133Cys [n = 1], p.Arg168* [n = 1], p.Arg255* [n = 1], p.Arg270* [n = 1], p.Arg294* [n = 4], p.Arg306Cys [n = 2], p.Thr158Met [n = 10] and other [6]). Assessments were conducted a median (IQR) of seven (7, 7) days apart. Reliability of the two tests for each of the total and subscale scores was strong and ICC (95%CI) values are shown in Table 5. The standard error of measurement was 1.5 for the total score and the minimal detectable difference was 4 points on the 45-point scale, indicating that an observed difference on the same individual of at least this magnitude would be necessary to be 95% confident that the difference was greater than measurement error. The standard error of measurement and minimal detectable difference values for the total and subscale scores are shown in Table 5.

**Table 5:** Test retest consistency and within subject measurement error for the total and subscale scores (N = 38).

	Intraclass correlation coefficient (95%CI)	Standard error of measurement^a^	Minimal detectable difference^b^
Total score (/45)	0.988 (0.978, 0.934)	1.5	4
Sitting subscale (/9)	0.920 (0.851, 0.957)	0.7	2
Standing and Walking subscale (/27)	0.983 (0.968, 0.991)	1.4	4
Challenge subscale (/9)	0.983 (0.969, 0.991)	0.3	1

^a^ Standard error of measurement is the square root of the mean square within subject error term using repeated measures analysis of variance.

^b^ Minimal detectable difference calculated as the SEM x 1.96 x √2.

## Discussion

We have extended our previous assessment of a 15-item gross motor assessment scale for Rett syndrome, and analysed its measurement properties in a larger sample of girls and women. Principal components analysis indicated a three factor structure comprising subscales in relation to sitting, standing, walking and more challenging motor skills, and the total score and each subscale demonstrated strong internal consistency. Mutations associated with a milder phenotype achieved better gross motor scores and overall, there was decline in total and subscale scores with increasing age. There was moderate to good agreement between observed and parent reported skills and use of parent reported data for a single item had minimal effect on scores. Repeatability of the measure in a clinical setting was excellent.

We previously used principal components analysis in an earlier study using data from the ARSD (n = 99) and derived two factors, one to calculate a general motor skills score and the other for more complex motor skills.[12] We have now replicated this analysis using our current larger dataset and found a three factor solution. The Eigenvalues for the factors Standing and Walking, and Challenge were greater than 1.0 and the Eigenvalue for Sitting was just under 1.0, but this latter value was clearly higher than the remaining factors and with strong factor loading, we interpreted the data to indicate the presence of three distinct motor profiles. Standing and Walking represented skills in relation to transitioning from sitting to standing, standing and walking, Challenge represented more difficult transition skills and running, and Sitting represented different methods of sitting. Conceptually, the Challenge and Sitting factors would be appropriate to address variation in those who are more mildly or severely affected and this supports the validity of a three factor solution.

Mutation type accounts for substantial variation in general severity in Rett syndrome.[4, 5, 17] Compared to those with a large deletion and taking into account the effect of age, those with the p.Arg133Cys, p.Arg294* or p.Arg306Cys had better gross motor skills as indicated by higher total scores and the Sitting and Standing and Walking subscale scores. These findings are consistent with the literature. For example, mutations such as the p.Arg133Cys, p.Arg294* or p.Arg306Cys mutations are generally associated with better functional abilities and milder clinical severity, whereas mutations such as the p.Arg270* and the large deletion are generally associated with poorer functional abilities and a more severe clinical severity.[4, 5, 17, 18] Additionally, we found that those with the p.Arg294* mutation had higher scores also for the Challenge subscale. These were the most difficult skills to achieve and for this group, performance in day to day settings would likely enable a much richer capacity to move independently and negotiate complexities within the environment. Predicted relationships between our new factor structure and mutation support the construct validity of the scale.

Age was also associated with gross motor function. Compared with early childhood and taking into account the effects of mutation type, motor scores were substantially reduced in those older than 19 years. The literature indicates that some girls with Rett syndrome will maintain the ability to walk when adults[8, 19] and this was observed in our dataset for the Standing and Walking subscale and total scores on account of the protective effect of a milder mutation. However, others had poorer skills when older,[6, 12] possibly related to the effects of neurological impairments such as bradykinesia[6] and progressive scoliosis. Unexpectedly, sitting scores declined significantly from the teenage years. Decline in sitting at an earlier age could relate also to the impairments of dystonia, bradykinesia and progressive scoliosis but conceivably, could relate to longer time spent sitting, often in a wheelchair with less opportunity for practice of gross motor activities. The alignment of our observations in relation to effects of age in the literature also supports the construct validity of the scale.

We previously assessed chance-corrected agreement for each of the gross motor items when video was assessed by two trained observers and we demonstrated substantial to excellent inter-rater reliability.[12] Comparing observed versus parent reported scores, chance-corrected agreement was poorer. For some items, the Kappa statistic was in lower half of the range for “moderate” agreement and therefore the lower bound of the (95%CI) borders were near the cutpoint between the values classified as “moderate” and “poor” agreement. This could be because parents have different interpretations of what each level of assistance comprised or there could be variability across a day or between days. We requested that video data were collected when the girls or women were well in their everyday settings and so we expect that the observed functional abilities were similar to what would be performed on a regular daily basis. These data highlight the need for clear and precise discussion with parents if estimating skill levels that have not been observed. There was little effect on summed scores when one item of parent report data was used, likely because some parents over- and others under-report their daughter’s skill levels. Therefore, regression can be used as a method of filling in missing values when assessing group data or parent reported data can be used in the calculation of scores if not every item can be observed during a clinical assessment. For total and subscale scores, the high Cronbach’s alpha values indicated strong internal consistency and the high ICC values indicated excellent repeatability. Our findings therefore provide multiple insights into the reliability of the RSGMS with favourable evidence for the internal consistency of the scale and stability of measurement. Finally, for any individual, we are confident that a change in the total score of 4 points would be greater than measurement error. During an assessment, this increase would identify individual improvement and could also be a feasible target when aiming to improve gross motor skills in Rett syndrome.

Rett syndrome is a rare disorder[2] but database infrastructure can be effective in the recruitment and analysis of large sample sizes[13]. The current study has accumulated gross motor data on 293 individuals by combining the resources of the ARSD with those of the multidisciplinary Danish Rett Syndrome Center. The development of well-validated measures is critical in our current era of clinical trials for neurodevelopmental disorders[20] and our data suggest that the RSGMS has potential to be useful in this regard. We acknowledge the limitation that not all skills were observed on every videoed assessment. However, three quarters had complete data and 10 or more of the 15 skills could be scored on more than 95% of videos. Principal components analysis using pairwise rather than listwise deletion allowed us to maximise the use of our data.[14] Additional analyses are necessary to continue building the case for the validation of the RSGMS. For example, future analyses could include relationships between scale scores and general clinical severity, the magnitude of scoliosis, longitudinal trajectories, and importantly assessment of responsiveness to change.

The RSGMS is a measure of everyday performance of gross motor skills for use in Rett syndrome. The measure is feasible to administer and allows assessment of important skills of everyday living. Using a large dataset, our study has allowed comprehensive evaluation of the validity and reliability of the RSGMS and we suggest it has a role to play in clinical monitoring and as an outcome measure in clinical trials.

## Supporting Information

S1 Appendix General instructions, item definitions and scoring for the Rett Syndrome Gross Motor Scale. (DOCX)

S1 Table Danish data. (DOCX)
