# Supplementary material for: Validating the Rett Syndrome Gross Motor Scale
Source: PLoS One. 2016 Jan 22;11(1):e0147555. doi: 10.1371/journal.pone.0147555 (PMC4723034; doi:10.1371/journal.pone.0147555)
Supplement: S1 Appendix — (DOCX) [file pone.0147555.s001.docx]

**S1 Appendix General instructions, item definitions and scoring for the Rett Syndrome Gross Motor Scale**

**General instructions:**

1. Assessment needs to be conducted when the child is well and not hungry or tired.
2. The assessment is observational and the parent caregiver/therapist can encourage each of the activities and provides assistance as necessary.
   1. Base the assessment on spontaneous activity as far as possible. Then ask the parent caregiver/therapist to show you how the girl performs each task. Use engaging activities to encourage the child to maintain balance, transition or move.
   2. If the task is performed with assistance, ask to see also if the task can be performed with less assistance.
   3. Observe/film each task for long enough so that adequate duration or number of steps can be observed to enable scoring.
3. Complete your rating of the *level of assistance needed* and comment on the assessment sheet.
   1. No assistance – no support needed *including without the use of arms*;
   2. Minimal assistance - with single arm propping, light touch or one hand held;
   3. Moderate assistance - needing trunk support or two hands held; and
   4. Maximal assistance - if support for upright posture, balance and movement is needed in order to accomplish the movement or if the child is unable to perform.

**Item definitions:**

1. Sitting on floor for 10 seconds
   1. The child can sit cross legged, in side sitting or in long sitting.
   2. Sit where you yourself can best support the child if necessary and consider having a second person who can provide encouragement from the front.
   3. Provide as much assistance as is necessary and observe sitting for at least 10 seconds.
2. Sitting on a chair with a back for 10 seconds
   1. Use an appropriately sized chair with a firm base and back (like a kitchen chair) that allows the child to sit with feet on the ground.
   2. Sit where you yourself can best support the child if necessary and consider having a second person who can provide encouragement from the front.
   3. Provide as much assistance as is necessary and observe sitting for at least 10 seconds.
3. Sitting on a stool for 10 seconds
   1. Can be on a stool or box, but again with a firm base. The child can sit forward on a chair with a back but not use the back of the chair for support.
   2. Sit where you yourself can best support the child if necessary and consider having a second person who can provide encouragement from the front.
   3. Provide as much assistance as is necessary and observe sitting for at least 10 seconds
4. Sit to stand
   1. Position the child in the same chair as for item b.
   2. Encourage the child to stand up.
   3. Stand where you yourself can best support the child if necessary and consider having a second person who can provide encouragement/an engaging activity from the front.
   4. Provide as much assistance as is necessary from light touch to trunk and pelvic support.
5. Standing three seconds
   1. The child should be positioned whilst standing comfortably on the floor (not a soft surface).
   2. Stand where you yourself can best support the child if necessary and consider having a second person who can provide encouragement/an engaging activity from the front.
   3. Provide as much assistance as is necessary from light touch to trunk and pelvic or trunk support.
   4. Film/observe for a period of time to include at least 3 secs whilst the feet are still – ie the child can adjust stance but not take a step in any direction.
6. Standing 10 seconds
   1. The child should be positioned whilst standing comfortably on the floor (not a soft surface).
   2. Stand where you yourself can best support the child if necessary and consider having a second person who can provide encouragement/an engaging activity from the front.
   3. Provide as much assistance as is necessary from light touch to trunk and pelvic support.
   4. Film/observe for a period of time to include at least 10 secs whilst the feet are still – ie the child can adjust stance but not take a step in any direction.
7. Standing 20 seconds
   1. The child should be positioned whilst standing comfortably on the floor (not a soft surface).
   2. Stand where you yourself can best support the child if necessary and consider having a second person who can provide encouragement/an engaging activity from the front.
   3. Provide as much assistance as is necessary from light touch to trunk and pelvic support.
   4. Film/observe for a period of time to include at least 20 secs whilst the feet are still – ie the child can adjust stance but not take a step in any direction
8. Walks 10 steps
   1. Encourage walking in a forward direction and consider having somebody or a favourite object in front of the child for motivation.
   2. One step is the forward movement of one leg from push off to heel strike.
   3. The steps must be consecutive with no more than a 2 or 3 second pause.
   4. Provide support as necessary.
   5. Film and observe for 10 steps that carry the body in a forward direction.
9. Side steps
   1. Walk towards a piece of furniture and then observe whether the girl will side step to change her forward direction.
   2. Provide support as necessary.
10. Turns 180 degrees
    1. Observe free walking activity or walk with the girl into a corner or and then encourage her to turn and walk in another direction. A turn of at least 90 degrees is required.
    2. Provide support as necessary.
11. Walks on a slope
    1. Use a minimum gradient of the slope of 1 in 10, and observe walking over several metres.
    2. Provide support as necessary.
12. Steps over an obstacle
    1. If there is no natural runner in a doorway, tape down an obstacle approximately 1 to 2 cm in height and about 2 to 3 cm wide across a doorway and observe whether the girl can step over the obstacle with foot clearance and free arms, or needs to hold the door frame or other assistance.
    2. Provide support as necessary.
13. Stands up from the floor
    1. When the child is sitting on the floor, encourage her to stand up with an activity that requires her to stand.
    2. Stand where you yourself can best support the child if necessary and consider having a second person who can provide encouragement/an engaging activity from the front.
    3. Provide as much assistance as is necessary from light touch to trunk and pelvic support.
14. Bending to touch the floor and returns to standing
    1. *The child must be able to stand without help to complete this item although could need support when performing the movement.*
    2. Put an important object on the floor (eg drink bottle, favourite toy) and encourage girl to reach down towards the floor and then stand back up again.
    3. Stand where you yourself can best support the child if necessary and consider having a second person who can provide encouragement/an engaging activity from the front.
    4. Provide as much assistance as is necessary from light touch to trunk and pelvic support.
15. Runs
    1. May need to run with the girl.

**Assessment form for the Rett Syndrome Gross Motor Scale**

| Item | Level of assistance | | | | Comments |
| --- | --- | --- | --- | --- | --- |
|  | Maximal  (0 points) | Moderate  (1 point) | Minimal  (2 points) | None  (3 points) |  |
| 1. Sitting on floor for 10 seconds |  |  |  |  |  |
| 2. Sitting on a chair with a back 10 seconds |  |  |  |  |  |
| 3. Sitting on a stool 10 seconds (no back support) |  |  |  |  |  |
| 4. Sit to stand |  |  |  |  |  |
| 5. Standing 3 seconds |  |  |  |  |  |
| 6. Standing 10 seconds |  |  |  |  |  |
| 7. Standing 20 seconds |  |  |  |  |  |
| 8. Walks 10 steps |  |  |  |  |  |
| 9. Side steps |  |  |  |  |  |
| 10. Turns |  |  |  |  |  |
| 11. Walks on a slope |  |  |  |  |  |
| 12. Steps over obstacle (height of a door frame) |  |  |  |  |  |
| 13. Stands up from the floor |  |  |  |  |  |
| 14. Bending to touch the floor and returns to standing |  |  |  |  |  |
| 15. Runs |  |  |  |  |  |

**SCORING**

|  | Items to sum | Score |
| --- | --- | --- |
| Total | All | ___ / 45 |
| Sitting subscale | 1, 2, 3 | ___ / 9 |
| Standing and Walking subscale | 4, 5, 6, 7, 10, 11, 12, 13, 14 | ___ / 27 |
| Challenge subscale | 8, 9, 15 | ___ / 9 |
