# Supplementary material for: Validating the Rett Syndrome Gross Motor Scale
Source: PLoS One. 2016 Jan 22;11(1):e0147555. doi: 10.1371/journal.pone.0147555 (PMC4723034; doi:10.1371/journal.pone.0147555)
Supplement: S1 Table — (DOCX) [file pone.0147555.s002.docx]

S1 Table Danish data

|  | | | | **Test 1** | | | | **Test 2** | | | |
| --- | --- | --- | --- | --- | --- | --- | --- | --- | --- | --- | --- |
| **Participant** | **Age** | **Mutation** | **Days between tests** | **Total** | **Sitting** | **Ambulation** | **Challenge** | **Total** | **Sitting** | **Ambulation** | **Challenge** |
| 1 | 3,95 | Other | 7 | 9 | 9 | 0 | 0 | 9 | 9 | 0 | 0 |
| 2 | 4,21 | Early truncating | 7 | 4 | 4 | 0 | 0 | 7 | 7 | 0 | 0 |
| 3 | 4,55 | p.R133C | 7 | 26 | 9 | 15 | 2 | 27 | 9 | 16 | 2 |
| 4 | 4,55 | Large deletion | 16 | 18 | 9 | 9 | 0 | 18 | 9 | 9 | 0 |
| 5 | 4,6 | p.T158M | 8 | 40 | 9 | 27 | 4 | 40 | 9 | 26 | 5 |
| 6 | 5,23 | p.R168X | 7 | 11 | 7 | 4 | 0 | 11 | 7 | 4 | 0 |
| 7 | 5,73 | p.R270X | 14 | 31 | 9 | 22 | 0 | 32 | 9 | 22 | 1 |
| 8 | 6,43 | p.T158M | 6 | 10 | 9 | 1 | 0 | 11 | 9 | 2 | 0 |
| 9 | 6,45 | p.T158M | 7 | 21 | 9 | 12 | 0 | 21 | 9 | 12 | 0 |
| 10 | 6,75 | Other | 7 | 9 | 9 | 0 | 0 | 9 | 9 | 0 | 0 |
| 11 | 7,18 | C-terminal | 7 | 42 | 9 | 27 | 6 | 41 | 9 | 26 | 6 |
| 12 | 8,07 | Other | 7 | 45 | 9 | 27 | 9 | 45 | 9 | 27 | 9 |
| 13 | 12,94 | Other | 7 | 16 | 9 | 7 | 0 | 19 | 9 | 10 | 0 |
| 14 | 14,39 | p.T158M | 6 | 2 | 2 | 0 | 0 | 2 | 2 | 0 | 0 |
| 15 | 14,58 | p.R106W | 7 | 1 | 1 | 0 | 0 | 1 | 1 | 0 | 0 |
| 16 | 14,91 | p.R294X | 8 | 40 | 9 | 25 | 6 | 40 | 9 | 25 | 6 |
| 17 | 15,12 | Large deletion | 6 | 24 | 6 | 18 | 0 | 24 | 6 | 18 | 0 |
| 18 | 15,55 | Other | 6 | 21 | 9 | 12 | 0 | 21 | 9 | 12 | 0 |
| 19 | 16,64 | p.R255X | 7 | 27 | 9 | 18 | 0 | 23 | 6 | 17 | 0 |
| 20 | 17,22 | C-terminal | 8 | 31 | 9 | 21 | 1 | 31 | 9 | 21 | 1 |
| 21 | 17,94 | Large deletion | 8 | 0 | 0 | 0 | 0 | 0 | 0 | 0 | 0 |
| 22 | 19,1 | pR294X | 20 | 45 | 9 | 27 | 9 | 45 | 9 | 27 | 9 |
| 23 | 19,56 | p.R294X | 7 | 6 | 6 | 0 | 0 | 6 | 6 | 0 | 0 |
| 24 | 20,25 | Early truncating | 20 | 6 | 6 | 0 | 0 | 6 | 6 | 0 | 0 |
| 25 | 21,66 | C-terminal | 7 | 40 | 9 | 27 | 4 | 38 | 9 | 25 | 4 |
| 26 | 27,44 | p.T158M | 7 | 42 | 9 | 27 | 6 | 42 | 9 | 27 | 6 |
| 27 | 33,12 | p.R306C | 7 | 28 | 9 | 18 | 1 | 28 | 9 | 19 | 0 |
| 28 | 34,04 | p.T158M | 7 | 34 | 9 | 24 | 1 | 30 | 9 | 20 | 1 |
| 29 | 34,66 | C-terminal | 7 | 38 | 9 | 27 | 2 | 40 | 9 | 27 | 4 |
| 30 | 36,04 | Early truncating | 7 | 23 | 9 | 14 | 0 | 23 | 6 | 17 | 0 |
| 31 | 36,59 | p.T158M | 7 | 31 | 9 | 21 | 1 | 26 | 7 | 18 | 1 |
| 32 | 36,81 | p.R306C | 6 | 38 | 9 | 27 | 2 | 38 | 9 | 27 | 2 |
| 33 | 43,43 | p.T158M | 6 | 20 | 9 | 10 | 1 | 28 | 9 | 18 | 1 |
| 34 | 44,09 | p.T158M | 7 | 17 | 6 | 11 | 0 | 17 | 7 | 9 | 1 |
| 35 | 44,99 | Large deletion | 18 | 9 | 9 | 0 | 0 | 9 | 9 | 0 | 0 |
| 36 | 54,83 | Other | 7 | 34 | 8 | 24 | 2 | 32 | 9 | 21 | 2 |
| 37 | 58,96 | p.R294X | 7 | 29 | 8 | 20 | 1 | 27 | 8 | 18 | 1 |
| 38 | 60,87 | p.T158M | 7 | 31 | 8 | 22 | 1 | 33 | 8 | 24 | 1 |
